# History of a Case of Abdominal Disease

**Published:** 1841-10

**Authors:** Wm. L. Sutton

**Affiliations:** Georgetown Ky.


ART. II.—*History of a case of Abdominal disease.* By WM. L. SUTTON, M. D., of Georgetown Ky.

Jacob Caplinger, aged 54, applied to me April 14th, 1838, to see if I could afford him any relief for a colic to which he was subject; and gave the following history of himself. He has been in bad health for several years, and is usually troubled with a costive state of the bowels. For the last year or two, he has had a tumor of considerable size in the left side of the abdomen. It commenced nearly equidistant between the navel and the short ribs; after remaining for some time, it disappeared from that place, and one was found in the left iliac region. At present it extends from the short ribs to near the pubis, where it terminates in an obtuse point, projecting to the median line. Just below the ribs is a point where there is an indistinct sense of fluctuation; bowels at present regular, but very subject to colic.

I did not see him again until the 20th of May, when he informed me that he had remained pretty much as when I saw him, until the 18th inst., when he was seized, at night, with considerable nausea and retching but no vomiting: he also belched up a considerable quantity of air. Soon after he fell asleep and enjoyed a tolerable night's rest, which he had not done for some time. The next morning he found the tumor had disappeared. Upon examination, I found the tongue tolerably clean, forward; but with a slimy fur towards the root; appetite indifferent, bowels very loose, dejections rhubarb-colored and very fœtid, urine high-colored, rather scanty and occasionally voided with great pain—respiration not oppressed—some cough—can sleep in a horizontal posture, which he had not been able to do for some time before the subsidence of the tumor; skin sallow and countenance indicative of suffering; abdomen somewhat distended and evidently containing a fluid; no appearance of the tumor, but, by pressing with some firmness in the left lumbar region, the edge of a solid body was evident, about two inches long; at this point there was considerable tenderness as well as over the abdomen generally; lancinating pains in the right hypochondrium; pulse not deranged. He had travelled about forty miles in a carriage on that day, which he bore badly.

From the 23d to the 27th he passed not a drop of urine, neither did he have the least inclination to do so; and, although there was no fulness in the region of the pubis, still to leave no room for doubt, on the 25th, at night, a catheter was introduced, but no fluid followed. During this time his bowels were getting into a better condition, both as to quality and quantity of the discharges. The skin was becoming of a better hue, the abdomen was diminishing in fulness; the tumor in the lumbar region increasing distinctly in size. The pulse continued about 65, and was of good volume; respiration easy; some cough, and expectoration of a little dirty looking mucus. On 27th he passed urine in a moderate quantity, apparently containing albumen, which continued in a fair quantity.

June 7th. Since the last report the abdominal tumor continued to increase in size until it extended from under the ribs considerably into the iliac region, where it terminated in a rounded point, very much as it did when I first examined it: laterally, it projected considerably beyond the navel, the surface presenting a lobulated character. The tumor was not very painful on pressure; but there was some tenderness in the epigastrium, and in the right hypochondriac region. Upon examining the abdomen on the 7th, the tumor had lost about three fourths of its size since the preceding day, with an increase of fluid in the abdomen. The intestinal evacuations were of a good color and consistence, but offensive in odor; urine natural in color and apparently containing albumen; pulse ranging from 65 to 70; respiration natural; sleeps well. June 29th. Had continued to improve until three days ago. His complexion had cleared up; pulse good, with an occasional intermission; bowels in good order, except that the dejections were offensive; appetite fair, and sleep in general comfortable. Three days ago he had a chill, which was said to have lasted two hours, after which there was some heat of the head and chest, with coolness of the hands and feet. Complexion became tawny, with an anxious expression of countenance; abdomen painful, and the next morning could not be moved without great pain, whereas he had sat up and walked about the house the best part of the previous day.— The bowels became loose; dejections light-colored and still more offensive. At present the equilibrium of the circulation is pretty well re-established; pain relieved, and sleep restored; but the bowels still deranged.

July 6th. Bowels have acted better; but the dejections still fœtid, and occasionally mixed with blood, (which comes from the lower part of the bowel,) and have been of proper color. Urine natural in color and quantity, without sediment. There is frequently considerable pain in the region of the bladder, on passing urine or fæces. At times complains of coldness of the right foot and leg. Pulse generally about 65, with an occasional intermission.

9th. The tumor for some days has had a very equivocal appearance. Lying in the track of the colon, its irregularities bear considerable resemblance to that bowel enormously distended: opposite the anterior superior spinous process of the ilium, it extends two-thirds of the way to the median line.

30th. Had continued to improve until a few days ago; his appetite was regular; digestion good; little eructation; fæces of good consistence, but still of an unnatural odor; skin rather dry; urine scanty; pulse 75 and pretty regular. Of late the tumor has increased in size and been more painful. The upper portion of the tumor extends across the epigastrium, and is uniform and hard; the lower portion reaches to the median line, and is uneven on its surface and softer, as if containing a fluid. Between these two portions is a depression. Lately, there has been an increase of fluid in the abdomen; also œdema of the feet. Yesterday, he was taken with an excruciating pain in the abdomen generally, and especially in the epigastrium, which was very tender to the touch, with inability to lie down for any length of time. [This inability to lie was occasioned by that posture causing great pain, and not by its oppressing respiration, which was then, and has been unaffected by position.] Lips blueish, and countenance expressive of great agony, yet the pulse continued firm. He had several discharges from his bowels, more soft and bilious than usual.

August 7th. There is less tenderness of the abdomen, yet there is considerable flatulence and pain in the bowels, just before the expulsion of the contents of the bladder or rectum; at the same time there is considerable pain in the glans penis. The upper portion of the abdomen is tympanitic, but the air is presumed to be in the stomach and bowels, as there is considerable eructation. The lower portion of the abdomen evidently contains a fluid. Pulse less intermittent, also less uniform in strength; skin somewhat clammy. The tumor not perceptible, except in the lumbar region, where it is of the size of a small fist.

15th. The tumor now occupies the iliac fossa, extending up as high as the ribs, which permit it to be followed by the hand, and across the epigastrium: there is a fissure between the upper and lower portion, the superior being smooth and the inferior lobulated, as on several occasions heretofore. Abdomen at present contains little fluid. Pressure on the back part of the lumbar region gives considerable pain, which is also felt in the shoulder. Bowels for a few days have been disposed to diarrhœa.

From this time until the 29th, on which day he died, he gradually sunk; the bowels being irregular, occasionally disposed to constipation, then to diarrhœa, attended with much pain in defecation; pulse fluctuating but never very frequent.

*Treatment.*— On the 20th of May when I commenced treating the case, my first effort was to bring the bowels into a tolerable condition. This was attempted by a mercurial cathartic, followed by calomel, ipecacuanha and opium in small doses, thrice a day. Under this course there was a gradual, but a very decided improvement, and, with the exception of short intervals towards the latter period of the disease, his bowels were in a very tolerble order, as to the quantity, consistence, and color of the discharges, which, however, always had an unnatural and offensive odor. For the suppression of urine, which lasted from the 23d to the 27th of May, various diuretics were tried, such as bals. copaib. and Venice turpentine, spts. nitr. dulc., and tr. canthar, &c., without any apparent effect. The secretion appeared to be re-instated by a blister over the kidneys. After this, and indeed, from the beginning, the treatment was varied to mitigate unpleasant symptoms, believing as I did, that the abdominal tumor was of the encysted kind and of course not to be reached by medical treatment.

The aggravation of symptoms which took place on the 29th of July, was caused by, or at least supervened very speedily upon, a brisk breeze accompanied by a heavy shower of rain, followed by a considerable fall of the thermometer, which had stood at 90.° He was at the time sitting between two doors, but he said there was not much of a current of air on him.

The following was my diagnosis, exhibited at the opening of the body, which was done in the presence of Drs. Barlow and Adams. "Abdomen containing a moderate quantity of turbid serum; the convolutions of the bowels attached by membranous adhesions; an encysted tumor occupying the left iliac, lumbar, and hypocondriac regions, pressing on the bladder and kidney; left lobe of the liver probably enlarged; mucous coat of intestines inflamed, perhaps exhibiting cicatrices of former minute ulcerations; kidneys diseased, perhaps, having a white deposite."

*Autopsy.*—Emaciation extrème; abdomen not prominent; omentum well down over the bowels, and adherent to them throughout, as also to the peritoneum; it and the bowels uniformly dark ; bowels very much agglutinated by membranous adhesions; abdomen contained from three quarts to a gallon of fluid, of the color of Port wine, which was confined in the lower region of the cavity, and below the bowels, by the adhesion of the omentum to the peritoneum and to the bowels, and by the intimate attachment of the convolutions of the intestines to each other; the bowels themselves of good calibre and empty. Occupying the upper part of the iliac region, and a considerable part of the lumbar region, was a tumor of the size of a child's head; in front, of cerebral consistence and color, while further back it was firm, but of the same hue. This tumor had a cyst in it, containing about a pint of fluid, in every respect like that contained in the cavity of the abdomen; its substance was the left kidney thus enormously enlarged and disorganized. Attached to the kidney, and apparently continuous with it, were one large and several small cysts, altogether containing about three pints of fluid. The large one held about a quart of fluid like that in the abdomen, mixed with considerable quantities of a substance resembling half putrid flesh. This mass extended from the pelvis to the diaphragm. The right kidney was rather pale and flaccid; the stomach contracted and apparently healthy; liver rather yellowish in color, of good size and appearance; spleen healthy.

### REMARKS.

I have been induced to give the foregoing free, and I fear, tedious extracts, from my memoranda of the case, because it is universally confessed that there is great difficulty in forming a correct judgment of the nature and seat of abdominal tumors; and because in these, as in other matters in our profession, the surest way to form a correct opinion is, to attend to and record the symptoms during life, and then by examination after death, ascertain the state of the parts. In this way, we can form a much better judgment of the correctness of our views and of the fitness of our treatment; we shall occasionally also be enabled to detect a cause of erroneous diagnosis.

The first interesting feature which I shall notice is the disappearance of the tumor on the 18th May, and its diminution at different times afterwards, followed by an increase of fluid in the abdomen, or rather its appearance there on the day above mentioned, for, upon my stating to him on the 20th that there was a quantity of water in the abdomen, he told me that a very respectable physician of Shelbyville, who had examined him on the 10th, (a few hours before he was attacked with nausea, &c.) told him explicitly that he had no fluid in the abdomen; and when I saw him in April, I certainly did not suspect him of being dropsical. Then this subsidence of the tumor and the effusion into the abdomen were clearly owing to a rupture of the walls of the sac. The sac was a thick, strong membrane, and after maceration in alcohol, exhibited a spot on the inner surface where there was an appearance of rupture, the inner layer being altogether shrunk to the size of a quarter of a dollar. Was the occurrence of nausea, retching, &c. on the night of 18th of May, the effect or cause of the discharge of fluid into the abdomen? or was it only a coincidence? Upon these points the patient could give no information, and every thing is left to conjecture. But the probability is, that it was only a coincidence, as no nausea, &c. attended the diminution which took place on the 7th June, and at other times, when I have little doubt that rupture of the sac took place.

2d. A total suppression of urine existed for four days, without his appearing to suffer any inconvenience. Not the least uneasiness or oppression of the brain.

3d. During this total suppression of urine, and whilst the intestinal evacuations were daily becoming less frequent, smaller, and more consistent, there was an evident diminution of the fluid in the abdomen, and that without any perceptible perspiration.

4th. The great pain, which was frequently felt upon discharging the contents of the bowels or bladder, most probably depended upon the tumor pressing on the parts concerned.

5th. The irregular knobbed-appearance which the tumor assumed towards the last, made me hesitate whether I had not mistaken the nature of the case, or at least whether there was not a vast accumulation of fæces in the sigmoid flexure of the colon as well as higher up. It was only by reflecting that no scybala were passed, and that there was a decided diminution of the tumor without any increased discharge from the bowels, that cleared away these doubts.

6th. The post-mortem examination very satisfactorily explained why the upper portion of the abdomen was tympanitic and the lower dropsical, inasmuch as the fluid was confined in the lower portion by a very complete adhesion of the omentum to the peritoneum, and to the convolutions of the intestines, aided by adhesions among the latter.

7th. It is worthy of remark, that the sac, distended with fluid, on several occasions, presented more resistance than the encephaloid mass. At one time at least, if I had attempted to draw off the fluid by puncture, I should have plunged the trocar into the soft tumor, and not into the sac.

And lastly, we may remark the speedy growth of the tumor. On the 20th May it was scarcely if at all perceptible, and on 29th August it was as large as a child's head.

It will be observed that no mention is made of the condition of the mucous coat of the intestines. The color of the bowels externally was so dark, that we were under the impression at the time, that gangrene had progressed too far to admit of their being satisfactorily examined. I soon after changed my opinion, and thought that the color of the bowels was imparted by the fluid which was in the abdomen, and I therefore very much regret the omission.

It is probable that the agglutination of the peritoneum, the omentum, and the convolutions of the bowels, was the consequence of inflammation produced on the 29th July, by the sudden change of weather. After that time the fluctuation was confined to the lower portion of the abdomen, whereas before, when there was a quantity of fluid in the abdomen, fluctuation was general.

*October*, 1840.

